# Indicator-based approach for fluvial flood risk assessment at municipal level in Slovakia

**DOI:** 10.1038/s41598-023-32239-7

**Published:** 2023-03-27

**Authors:** Matej Vojtek

**Affiliations:** 1 Department of Geography, Geoinformatics and Regional Development, Faculty of Natural Sciences and Informatics, Constantine the Philosopher University in Nitra, Trieda A. Hlinku 1, 949 01 Nitra, Slovakia; 2 Institute of Geography, Slovak Academy of Sciences, Štefánikova 49, 814 73 Bratislava, Slovakia

**Keywords:** Hydrology, Natural hazards

## Abstract

The article focuses on the mapping and assessment of fluvial flood risk at municipal level of Slovakia. The fluvial floods risk index (FFRI), composed of a hazard component and a vulnerability component, was computed for 2927 municipalities using spatial multicriteria analysis and geographic information systems (GIS). The fluvial flood hazard index (FFHI) was computed based on eight physical-geographical indicators and land cover representing the riverine flood potential and also the frequency of flood events in individual municipalities. The fluvial flood vulnerability index (FFVI) was calculated using seven indicators representing the economic and social vulnerability of municipalities. All of the indicators were normalized and weighted using the rank sum method. By aggregating the weighted indicators, we obtained the FFHI and FFVI in each municipality. The final FFRI is a result of a synthesis of the FFHI and FFVI. The results of this study can be used mainly in the framework of flood risk management at national spatial scale, but also for local governments and periodic update of the Preliminary Flood Risk Assessment document, which is carried out at the national level under the EU Floods Directive.

## Introduction

Due to the climate change effects, also a change in the frequency and intensity of storm surges, or long-lasting rainfall, which is the cause of different types of floods, such as fluvial, flash or pluvial, is expected^1–3^. Socio-economic development and related landscape changes have caused that the level of flood risk varies with space and time^4^. For these reasons, it is necessary to elaborate a comprehensive methodology for mapping and assessment of flood risk. In recent decades, an integrated approach based on flood adaptation, flood risk reduction, and flood mitigation has begun to be emphasized, which is contrary to an approach focused only on flood control through technical flood prevention measures^5–7^.

The basic step within the integrated approach to flood risk management is the identification of flood risk on the basis of which the selection of strategies for risk reduction can take place. Integrated flood risk assessment is based on a multidimensional definition of flood risk, i.e. flood damage is influenced not only by the probability of the occurrence of a particular flood scenario, but also by the vulnerability of the social, economic, and environmental system^8,9^. Flood risk can be expressed in absolute numbers, such as the expected monetary amount of damage. In this case, the vulnerability assessment is mostly dependent on the flood hazard^10^. The second method of flood risk assessment is based on its relative expression, while the use of multicriteria analysis methods has been proven in many works^11,12^. In this case, the vulnerability assessment is independent of the flood hazard and the defined indicators are aggregated into the vulnerability index^13–16^. There are various multicriteria analysis techniques for flood risk mapping, which were reviewed in more detail, for example, by Abdulrahman and Bwambale^17^ and practically applied in a number of studies, such as Kumar and Jha^18^, Shivaprasad Sharma et al.^19^, and so on.

In literature, the topic of flood risk mapping at municipal or national level was studied, for example, by Santos et al.^20^ in Portugal or Quesada-Román^21^ in Costa Rica, Tate et al.^22^ and Wing et al.^23^ in the US. Flood risk at regional level, either on the catchment or administrative scale, was studied, for example, by Roder and Sofia^24^, Wang et al.^25^, Mohanty et al.^26^, Pathak et al.^27^, Sajjad et al.^28^ or Tang et al.^29^. In case of Slovakia, a comprehensive assessment of flood hazard, flood vulnerability or flood risk at municipal level was performed in very few works. In particular, Solín et al.^9^ assessed flood risk, Solín^30^ assessed flood hazard, and Solín^31^ assessed flood vulnerability in municipalities located in headwater basins. At regional level, a comprehensive assessment of flood risk is provided by Solín and Rusnák^32^. The basic experience from the previously mentioned studies is that they mostly used the indicator-based and/or multicriteria approaches and methods for assessing the flood risk or its components: hazard and vulnerability. These approaches were used and tested in different study areas across the world and from our point of view, they are considered inevitable for assessing the flood risk at municipal level.

However, flood risk mapping and assessment at the level of all municipalities in Slovakia is still absent. The use of municipal level for flood risk assessment is based on the fact that each municipality can influence various aspects of prevention, mitigation, protection or adaptation to floods by managing its own territory and can use several tools for these purposes, such as spatial plans, grants for technical and non-technical flood measures, involvement of local residents and stakeholders in flood risk management, etc.^33–36^. When comparing the catchment level with the municipal level for flood risk assessment, we can see that both types are used in literature. The advantage of using catchment level is especially for flood hazard assessment and the associated runoff processes and connectivity^27^, which are “interrupted” when using the municipal level. However, many studies have used municipal level also for flood hazard assessment^26,29^ in order to combine it easily with the vulnerability assessment. As for the flood vulnerability assessment, it is more convenient to use municipal level since most of the relevant data are based on the census, which is carried out at the municipal (administrative) level^20,24,37,38^.

The advantage can be also seen in the effective use of the results by the bodies responsible for flood risk management and for the periodic update of the Preliminary Flood Risk Assessment (PFRA) in accordance with the EU Directive 2007/60/EC on the assessment and management of flood risks. According to Solín^39^, the methodology applied in PFRA^40,41^ documents to determine the so-called areas with potentially significant flood risk, including potentially significantly endangered municipalities, and critical river sections lacks conceptual basis without any clarifications of these areas or river sections. Therefore, the motivation of this study is to propose a comprehensive approach and new concept for possible updating of the PFRA by determining the fluvial flood risk index (FFRI) for all municipalities in Slovakia.

All in all, the aim of this study is to map and assess the fluvial flood risk at municipal level of Slovakia and determine the FFRI for 2927 municipalities. The calculated FFRI represents a synthesis of the fluvial flood hazard index (FFHI) and the fluvial flood vulnerability index (FFVI), which were computed with the use of spatial multicriteria analysis and geographic information systems (GIS).

## Research area

**Figure 1:**
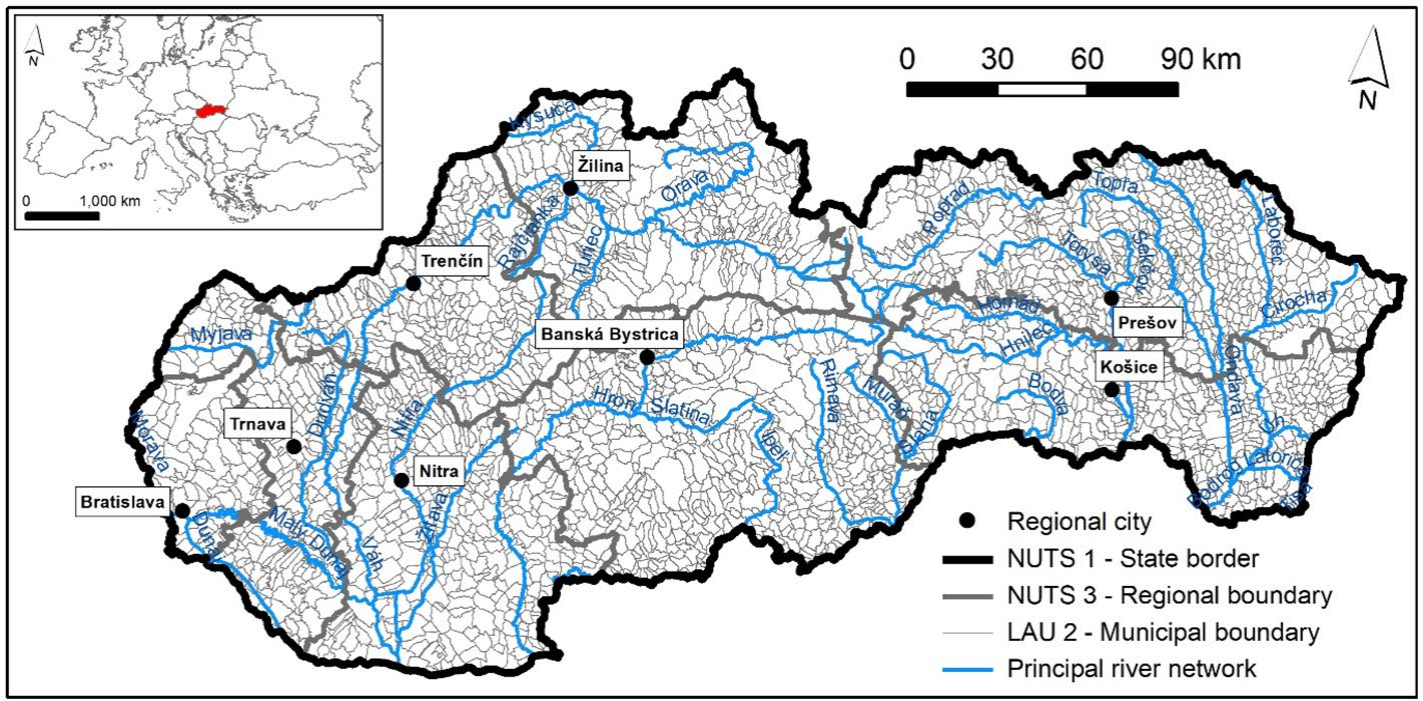
Research area: Slovakia—2927 municipalities (LAU 2 level). This figure was generated in ArcGIS 10.2.2 software.

Slovakia, representing the research area in this study, is a country in Central Europe with an area of 49,034 km^2^. Using the Nomenclature of Territorial Units for Statistics (NUTS), Slovakia is divided into one NUTS 1 region, four NUTS 2 regions, eight NUTS 3 regions, 79 LAU 1—Local administrative units (NUTS 4) regions, and 2927 LAU 2—Local administrative units (NUTS 5). In this study, we focused on the LAU 2 level, which is represented by 2927 municipalities (Fig. 1).

The main geological formations of Slovakia include the following: Cretaceous and Palaeogene of the Outer Carpathians (13.9%), Early Paleozoic—Proterozoic of the Veporicum and Tatricum (2.8%), Early Paleozoic of the Gemericum (2.3%), Late Cretaceous and Paleogene of the Inner Carpathians (10.4%), Late Paleozoic of the inner Carpathians (2.5%), magmatic rocks (5.2%), Mesozoic and Paleogene of the Klippen belt (2.7%), Mesozoic of the Inner Carpathians (12.1%), Neogene (37.3%), and Neogene volcanic rocks (10.9%).

The geomorphological units of Slovakia belong to the Alpine-Himalayan System, particularly the Carpathians sub-system represented by mountains and the Pannonian basin sub-system represented by lowlands and low-lying basins. The lowest elevation point has 94 m a.s.l. and is located in the Klin nad Bodrogom municipality (eastern Slovakia) while the highest point is the Gerlachovský štít (peak) with an elevation of 2655 m a.s.l. and it is located in the Tatra Mts. Maximum relief is 2561 m. Approximately 41% of Slovakia lies in altitudes < 300 m a.s.l., 45% of the research area lies in altitudes between 300 and 800 m a.s.l., 13% of the territory is located in altitudes between 800 and 1500 m a.s.l., and less than 1% of Slovakia lies in altitudes between 1500 and 2655 m a.s.l. According to the climatic classification by Lapin et al.^42^, Slovakia belongs to three climatic regions: warm, moderately warm, and cool region.

Most of the Slovak rivers flow into the Danube River with approximately 96% of all waters draining into the Black Sea. The rest of waters flow into the Poprad and Dunajec rivers and is drained into the Baltic Sea. According to Šimo and Zaťko^43^, Slovak rivers are characterized by three types of runoff regime: temporary snow, snow-rain combined, and rain-snow combined.

The share of the main land use/land cover (LULC) classes for the year 2021 is the following (Statistical Office of the Slovak Republic, 2023): water area (1.9%), forests (40.4%), urban, industrial, and other area (20.8%), arable land (26.2%), permanent crops (0.3%), and permanent meadows and pastures (10.4%).

## Methodology

**Figure 2:**
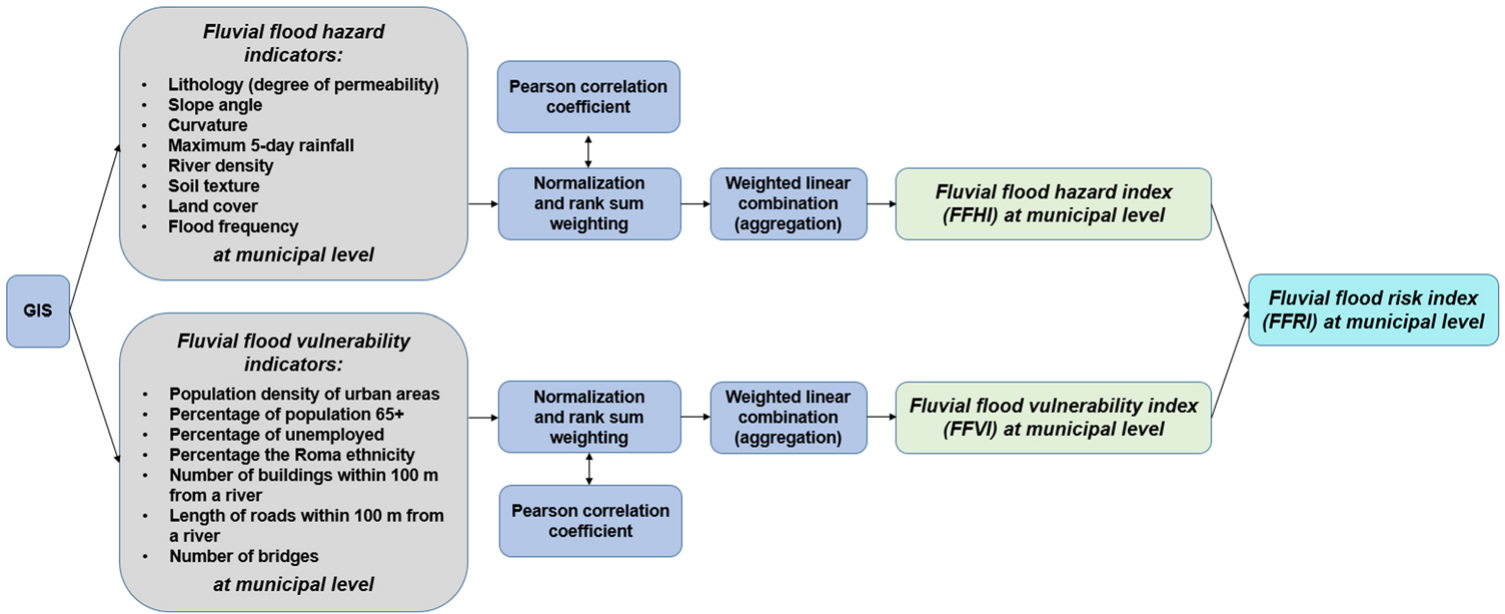
Methodological approach used in this study.

The overall methodology applied in this study is graphically presented in Fig. 2. The methodological approach is divided into the following main steps: (1) selection and processing of flood hazard indicators and flood vulnerability indicators using ArcGIS 10.2.2 software; (2) normalization of hazard and vulnerability indicators using the min–max method; (3) calculating the Pearson correlation coefficient for determining the importance of indicators; (4) weighting of indicators using the rank sum method; (5) weighted linear combination (aggregation) of indicators into the fluvial flood hazard index (FFHI) and fluvial flood vulnerability index (FFVI); and (6) multiplication of FFHI and FFVI into the final fluvial flood risk index (FFRI). The next sub-Sects. 3.1 and 3.2 describe the data and methods used in this study.

### Data

#### Fluvial flood hazard indicators

Altogether, we chose eight relevant indicators for determining the FFHI: lithology, slope angle, curvature, 5-day maximum rainfall, river density, soil texture, land cover, and number of flood events in municipalities. The selected hazard indicators are considered relevant, based on the author's experience, and important for fluvial flood hazard mapping, which is evidenced in similar research performed not only in Slovakia^9,32^, but also in other countries^9,20,21,27,44^. All hazard indicators were processed as the vector GIS layers, i.e. data in raster format (i.e. slope and curvature) were converted to vector layers using the ArcGIS 10.2.2 software.

Lithology indicator, i.e. degree of permeability of individual rock types, was based on the Map of Engineering Geological Zones at a scale of 1:50,000, which is available at the websites of State Geological Institute of Dionýz Štúr in Bratislava, and the work of Hrnčiarová^45^. The indicator of the number of flood events by municipalities was prepared from the following sources for the years 1997–2021: (1) Reports on the Course and Consequences of Floods in the Slovak Republic available from 2001 at the website of the Ministry of Environment of the Slovak Republic and (2) documents of the Preliminary Flood Risk Assessment in the Slovak Republic^40,41^ available at the websites of the Ministry of Environment of the Slovak Republic. This indicator was included among other flood hazard indicators in order to differentiate the fluvial flood hazard among the municipalities. For instance, a municipality can have very high or high fluvial flood hazard due to physical-geographical indicators and land cover, but with no occurrence of flood events. On the other hand, a municipality can have very low or low fluvial flood hazard due to physical-geographical indicators and land cover, but with some occurrence of flood events. Due to these reasons, we included the flood frequency as an indicator, implying that the fluvial flood hazard is higher with the increasing number of flood events. In addition, the flood frequency was used as an indicator in other similar studies, for example, Solín and Rusnák^32^ or Solín et al.^9^.

Source data for calculating the slope and curvature indicators was represented by a digital elevation model (DEM) with the resolution of 10 m (DMR3.5) available from the website of the Geodetic and Cartographic Institute in Bratislava.

As for the 5-day maximum rainfall, this map was digitized in GIS based on the original map of average annual maximum values of 5-day rainfall presented in the Climate Atlas of Slovakia^46^, which was created from daily values of rainfall at the available rainfall stations during the period 1981–2010 using the geographically weighted regression. The river density indicator was calculated with the use of a GIS layer of river network compatible to the DEM and available at the website of the Geodetic and Cartographic Institute in Bratislava.

The soil texture indicator was calculated from two types of source data, both at a scale of 1:10,000. In particular, we used Evaluated soil-ecological units (BPEJ) and forest soil units. For deriving the land cover indicator, we used the latest CORINE Land Cover (CLC) vector database (2018). The source data for processing the hazard indicators are presented in Supplementary Table S1.

#### Fluvial flood vulnerability indicators

For determining the FFVI, we chose seven indicators, which are related to the economic and social vulnerability of assets and people to fluvial floods. In particular, it was the (1) population density of urban areas of municipalities, (2) share of population included in the age category 65+ from the total population of municipality, (3) share of unemployed persons from the total number of economically active population in municipality, (4) share of the Roma ethnicity from the total population of municipality, (5) the number of buildings within 100 m from a river, (6) length of roads within 100 m from a river, and (7) number of bridges in municipality. The selected vulnerability indicators were chosen based on the author's experience and literature review of similar research performed not only in Slovakia^31,32,47^, but also in other countries^20,21,24,37^.

The indicators of the economic vulnerability, i.e. susceptibility to possible damage by fluvial floods, are represented by the number of buildings within 100 m from a river, length of roads within 100 m from a river, and the number of bridges, which were processed based on the Basic Data Base for the Geographic Information System (ZBGIS), which is provided by the Geodetic and Cartographic Institute in Bratislava. Input data for another three indicators representing the social vulnerability (population density of urban areas, population category 65+, and unemployed) were retrieved from the Data cubes available at the websites of the Statistical Office of the Slovak Republic. Regarding the population category 65+, it is the most vulnerable age category because of declining physical strength and mobility and also many of these people live alone. Furthermore, the unemployed persons have increased vulnerability due to their low or no income and possible problems with recovering from a flood event. The last indicator of the share of Roma ethnicity from the total population in municipality was prepared from the source data presented in the Atlas of Roma Communities (2019)^48^. This indicator was chosen due increased vulnerability of the Roma ethnicity, which concerns mainly the construction of colonies near water streams, poor quality of dwellings, like huts and shacks, and their overcrowding^31^. The source data for processing the vulnerability indicators are presented in Supplementary Table S1.

### Spatial multicriteria analysis

#### Normalization and weighting of indicators

Regarding the hazard indicators, we followed the methodology presented in the study by Vojtek et al.^49^. First, six hazard indicators (i.e. lithology, slope, curvature, 5-day maximum rainfall, soil texture, and land cover) were classified into categories or intervals. The division of six hazard indicators into the classes is presented in Supplementary Table S2. After that, we ranked the importance of each class of these six indicators (see Table S2), where 1 was the most important class. The next step was to calculate the share of each class of these six indicators on the extent of the municipality using GIS. Then, we multiplied the share of the class with corresponding weight, which was calculated using the rank sum method (Eq. 1)^50^:

1 $${w}_{j}=\frac{n-{r}_{j}+1}{\sum_{k=1}^{n}n-{r}_{k}+1}$$

where *w*_*j*_ is the normalized weight for the *j*-th indicator class; *n* is the number of indicator classes under consideration; and *r*_*j*_ is the rank position of the *j*-th indicator class, as presented in Vojtek et al.^49^. In the next step, the weighted classes of each of the six hazard indicators were summed in order to have one quantitative value for each indicator and then normalized to the scale [0, 1] using the min–max method.

Furthermore, the rest of the hazard indicators (i.e. river density and number of flood events) contain quantitative data, which were not classified into intervals and, therefore, they were directly normalized to the range [0, 1] using the min–max method.

As long as all of the vulnerability indicators also contain quantitative data, they were directly normalized to the scale [0, 1] using the min–max method.

To calculate the weights of hazard and vulnerability indicators, we first used the Pearson correlation coefficient to determine the strength of correlation between each normalized indicator and normalized number of flood events in municipality. The reason for choosing the Pearson correlation coefficient was to find out the relation between individual hazard/vulnerability indicators and the number of flood events, which occurred in municipalities between 1997 and 2021. Using the values of Pearson correlation, we ranked the indicators from the most important (the highest strength of Pearson correlation) to the least important (the lowest strength of Pearson correlation). Based on the rank sum method (Eq. 1), we calculated the final normalized weights of each indicator. The reason for choosing the rank sum method is that it has an explanatory power when a reasonable number of indicators is used. As long as the number of FFHI indicators is eight and the number of FFVI indicators is seven, the individual weights are differentiated enough, i.e. the difference among the values of indicator weights is big enough. If too many indicators are used, some of the weights can have very similar values^51^.

#### Aggregation of indicators and determination of FFRI

The FFHI and FFVI were calculated based on the weighted linear combination technique, where individual indicators were multiplied with their corresponding normalized weights and then aggregated into the respective index. The following Eq. (2) was used to calculated the FFHI and FFVI^52^:

2 $$FFHI{\text{/}}FFVI = \sum\limits_{j} {x_{j}^{\prime } w_{j} }$$

where *FFHI* and *FFVI* are the fluvial flood hazard index and fluvial flood vulnerability index, respectively; *w*_*j*_ is the normalized weight of the *j*-th indicator; and *x′*_*j*_ is the *j*-th indicator in the range [0, 1].

The final FFRI for each municipality was determined as the product of FFHI and FFVI indices based on Eq. (3):

3 $$FFRI=FFHI\times FFVI$$

where *FFRI* is the fluvial flood risk index; *FFHI* is the fluvial flood hazard index; and *FFVI* is the fluvial flood vulnerability index.

## Results and discussion

### Fluvial flood hazard indicators

**Figure 3:**
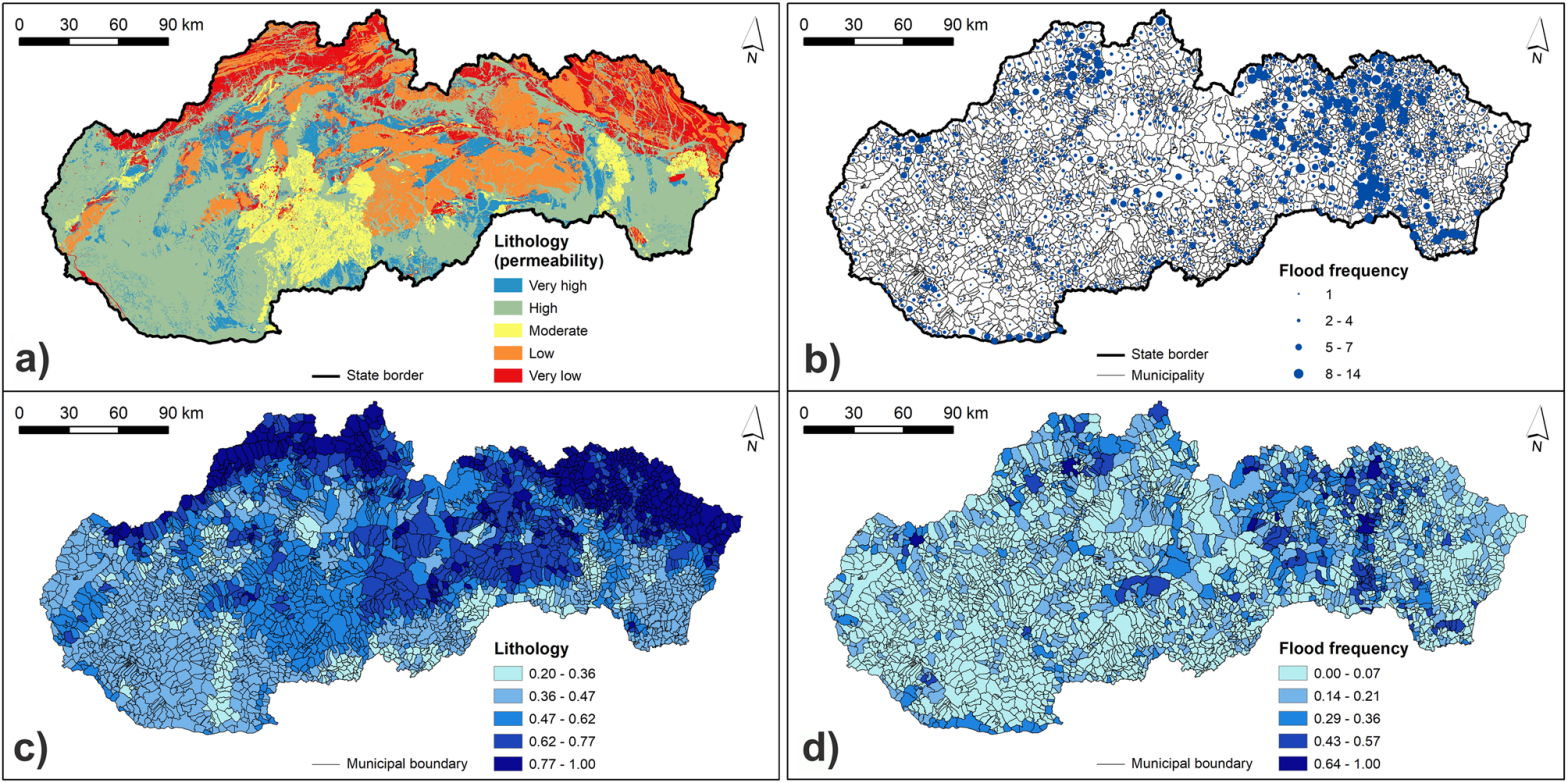
Lithology and number of flood events indicators: (**a**) original lithology values; (**b**) original flood frequency values; (**c**) normalized lithology values; (**d**) normalized flood frequency values. This figure was generated in ArcGIS 10.2.2 software.

**Figure 4:**
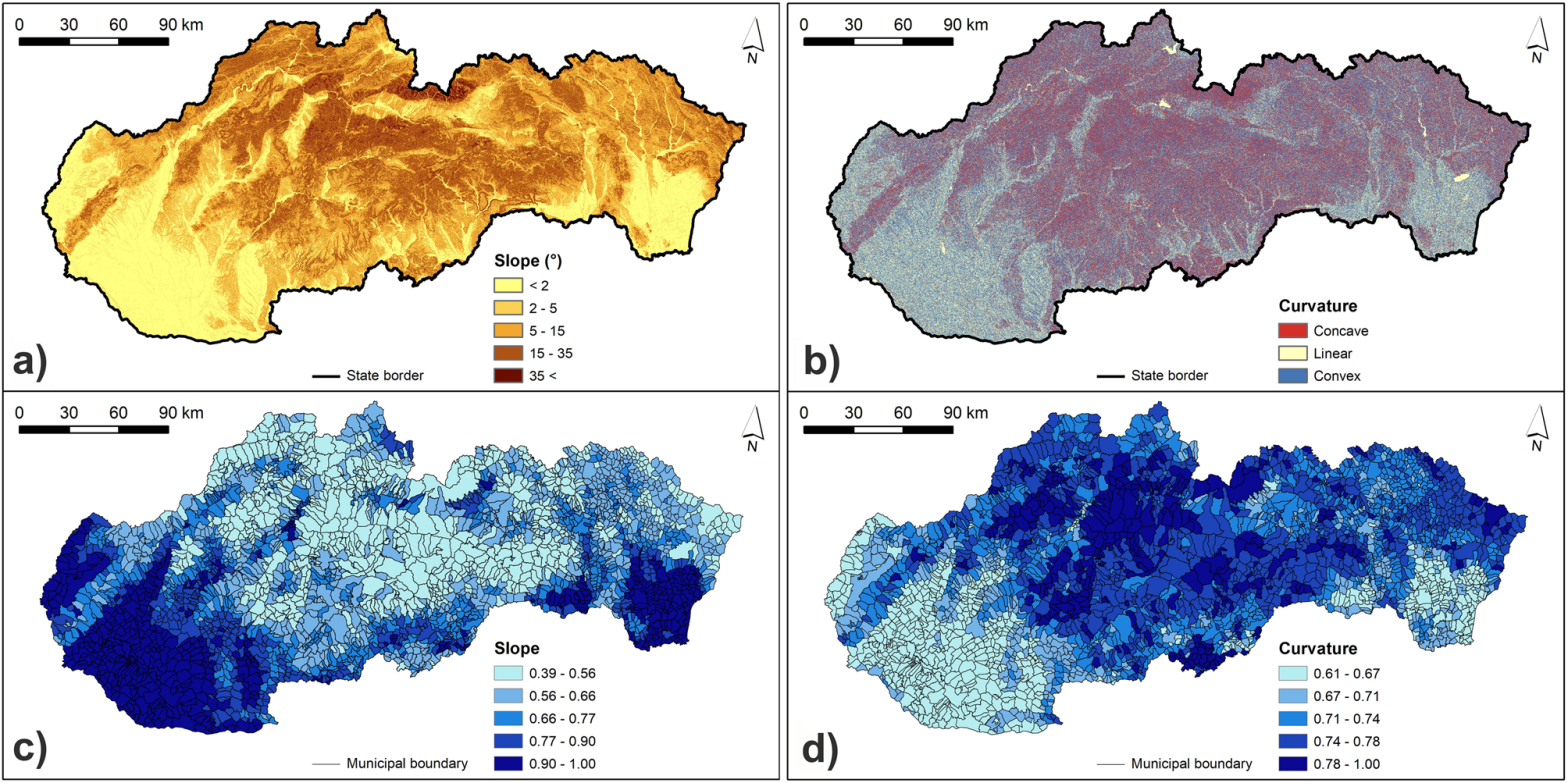
Slope and curvature indicators: (**a**) original slope values; (**b**) original curvature values; (**c**) normalized slope values; (**d**) normalized curvature values. This figure was generated in ArcGIS 10.2.2 software.

**Figure 5:**
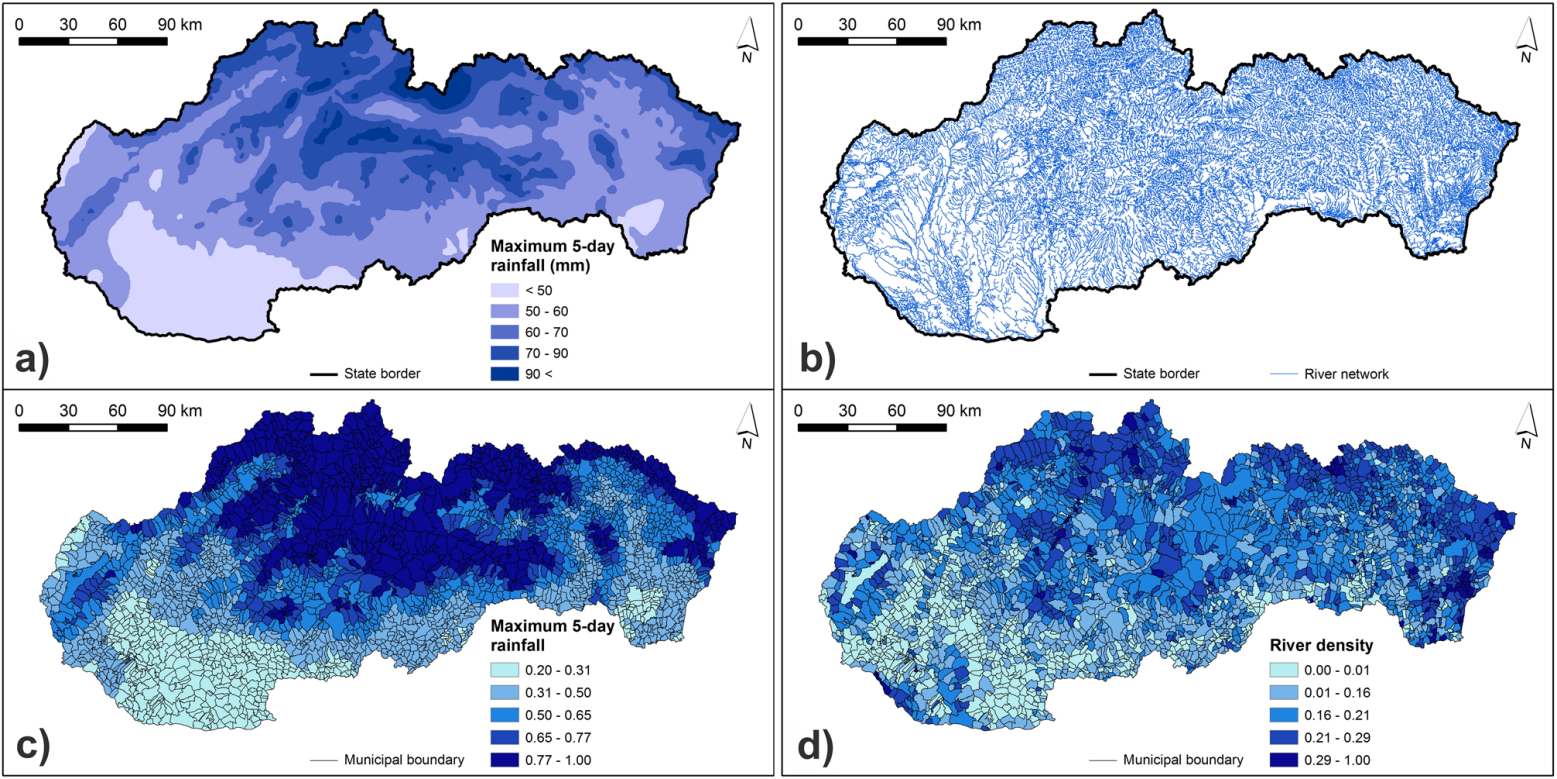
5-day maximum rainfall and river density indicators: (**a**) original 5-day maximum rainfall values; (**b**) river network; (**c**) normalized 5-day maximum rainfall values; (**d**) normalized river density values. This figure was generated in ArcGIS 10.2.2 software.

**Figure 6:**
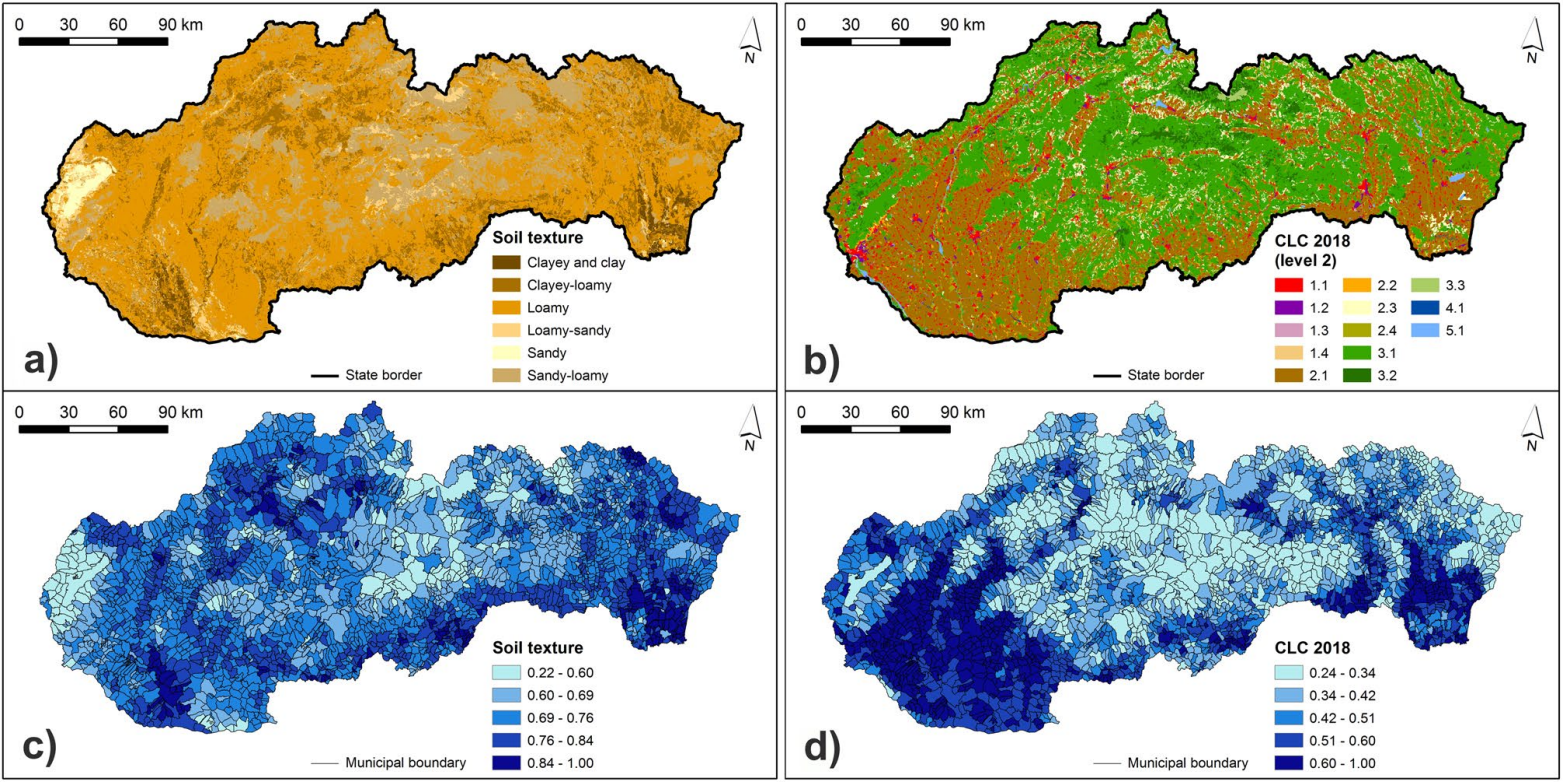
Soil texture and land cover indicators: (**a**) original soil texture values; (**b**) original land cover values; (**c**) normalized soil texture values; (**d**) normalized land cover values. This figure was generated in ArcGIS 10.2.2 software.

The calculated normalized class weights of hazard indicators are presented in Supplementary Table S2. Figures 3, 4, 5 and 6 present the original and normalized values of hazard indicators. Figure 3 shows the original values of lithology and flood frequency indicators. The lowest rock permeability and the highest number of flood events can be seen in the northern and north-eastern Slovakia. Figure 4 presents the slope values, which are the highest in municipalities of southern, south-western, and south-eastern Slovakia. Furthermore, this figure shows that the share of concave forms of terrain dominate in municipalities of central and also north-eastern Slovakia. Figure 5 shows that the highest values of 5-day maximum rainfall correspond to the occurrence of the highest mountains in Slovakia while the river density is the highest mainly in northern and eastern Slovakia. Figure 6 presents soil texture and land cover indicators. As for the soil texture, the municipalities with the lowest soil infiltration capabilities are located in several differently sized clusters, for example, in southern Slovakia. The interception capacity of land cover is the lowest mainly in south-western and south-eastern Slovakia, where the arable land dominates.

### Fluvial flood vulnerability indicators

**Figure 7:**
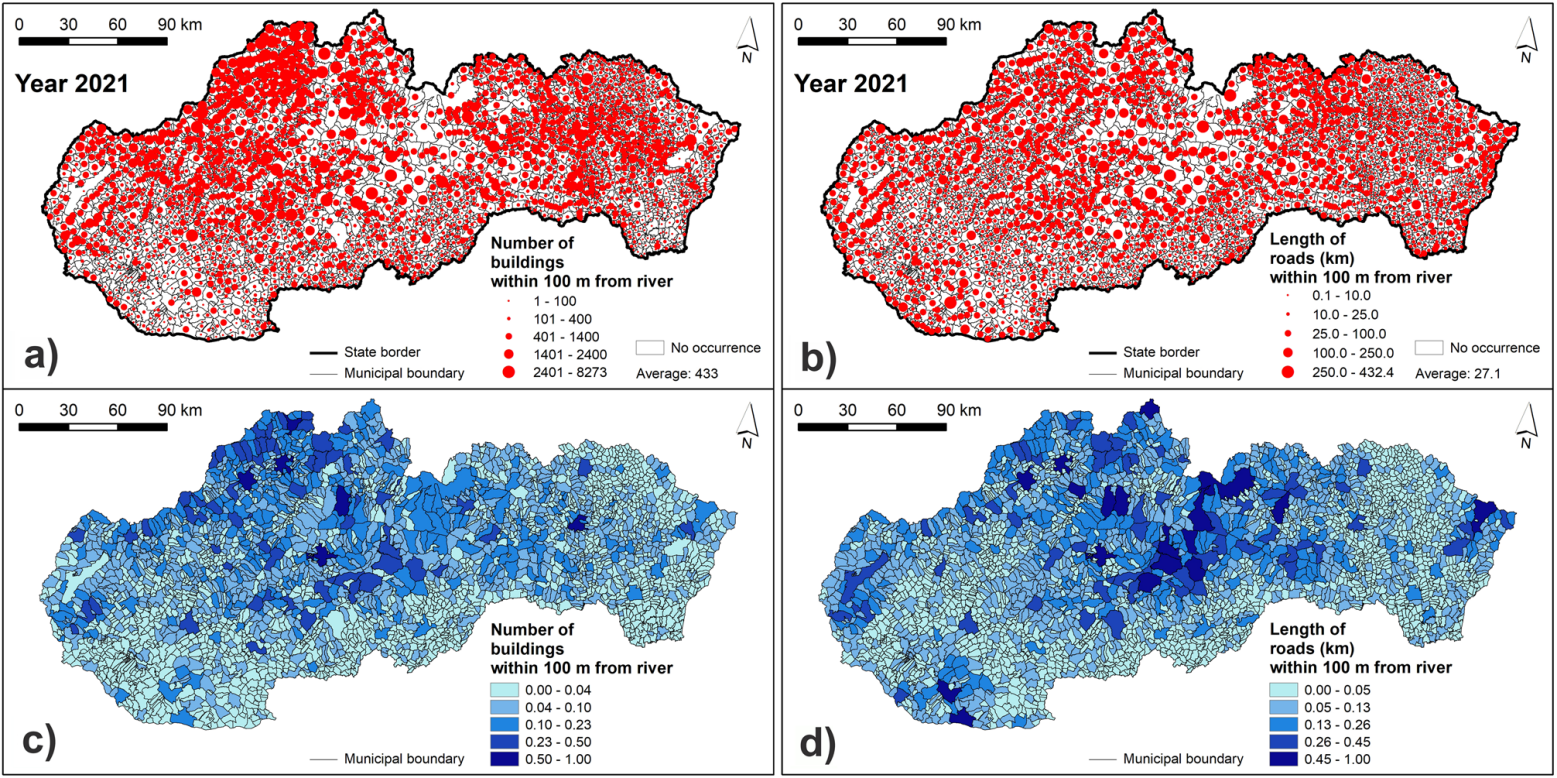
Indicators of the number of buildings and length of roads within 100 m from a river: (**a**) original values of the number of buildings within 100 m from a river; (**b**) original values of the length of roads within 100 m from river; (**c**) normalized values of the number of buildings within 100 m from a river; (**d**) normalized values of the length of roads within 100 m from a river. This figure was generated in ArcGIS 10.2.2 software.

**Figure 8:**
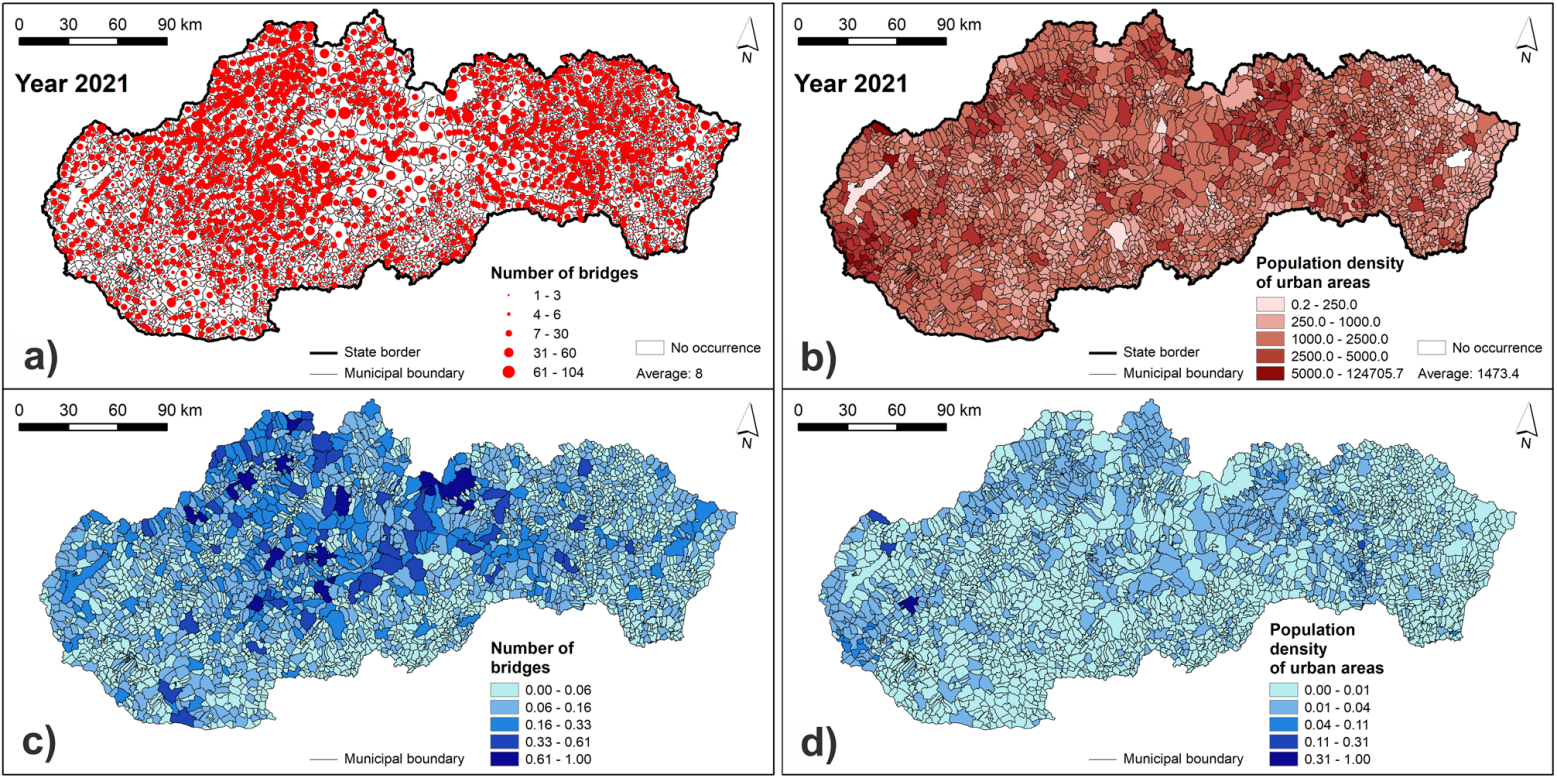
Indicators of the number of bridges and population density of urban areas: (**a**) original values of the number of bridges; (**b**) original values of the population density of urban areas; (**c**) normalized values of the number of bridges; (**d**) normalized values of the population density of urban areas. This figure was generated in ArcGIS 10.2.2 software.

**Figure 9:**
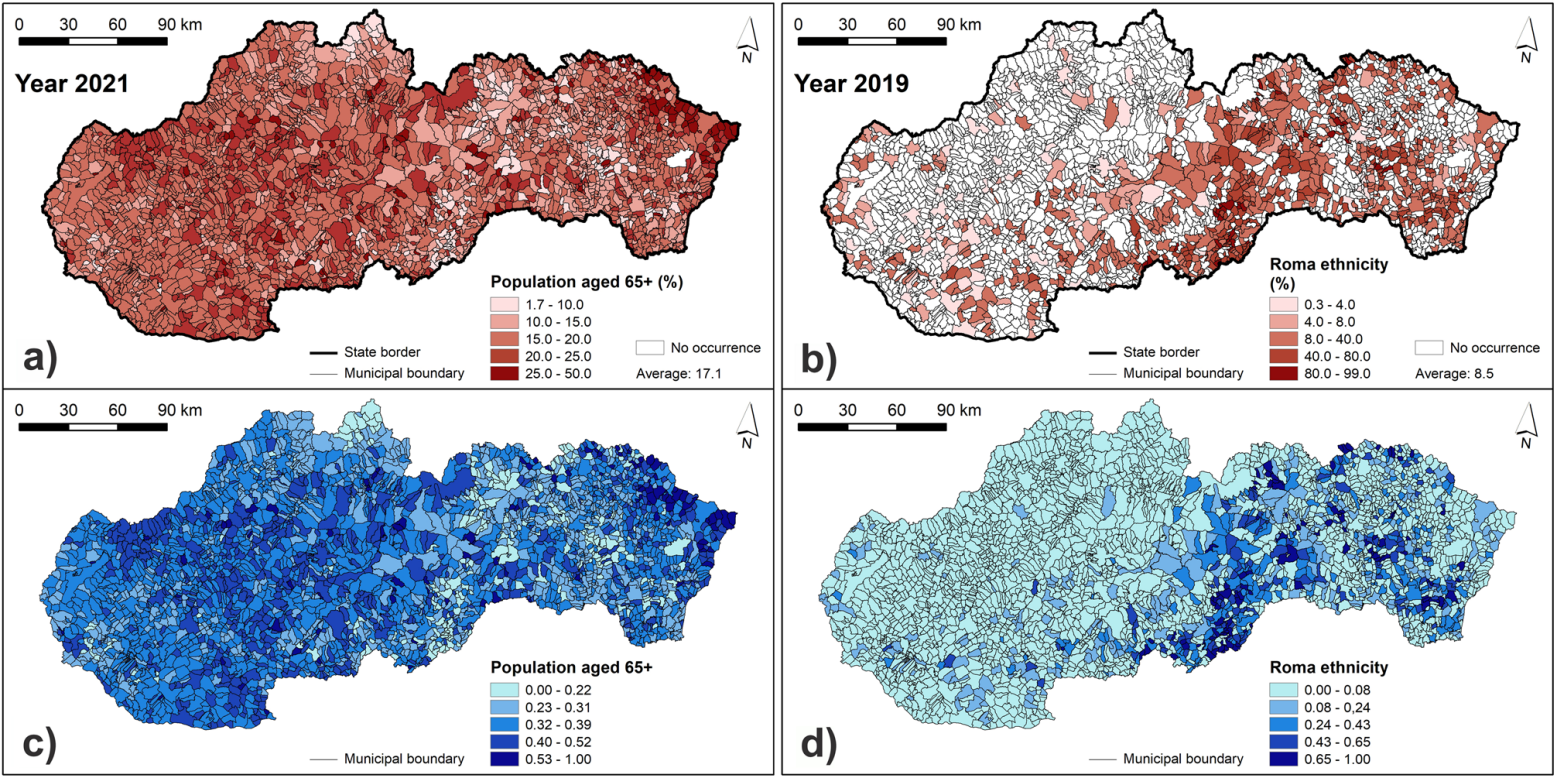
Indicators of the population aged 65+ and the Roma ethnicity: (**a**) original values of the population aged 65+; (**b**) original values of the Roma ethnicity; (**c**) normalized values of the population aged 65+; (**d**) normalized values of the Roma ethnicity. This figure was generated in ArcGIS 10.2.2 software.

**Figure 10:**
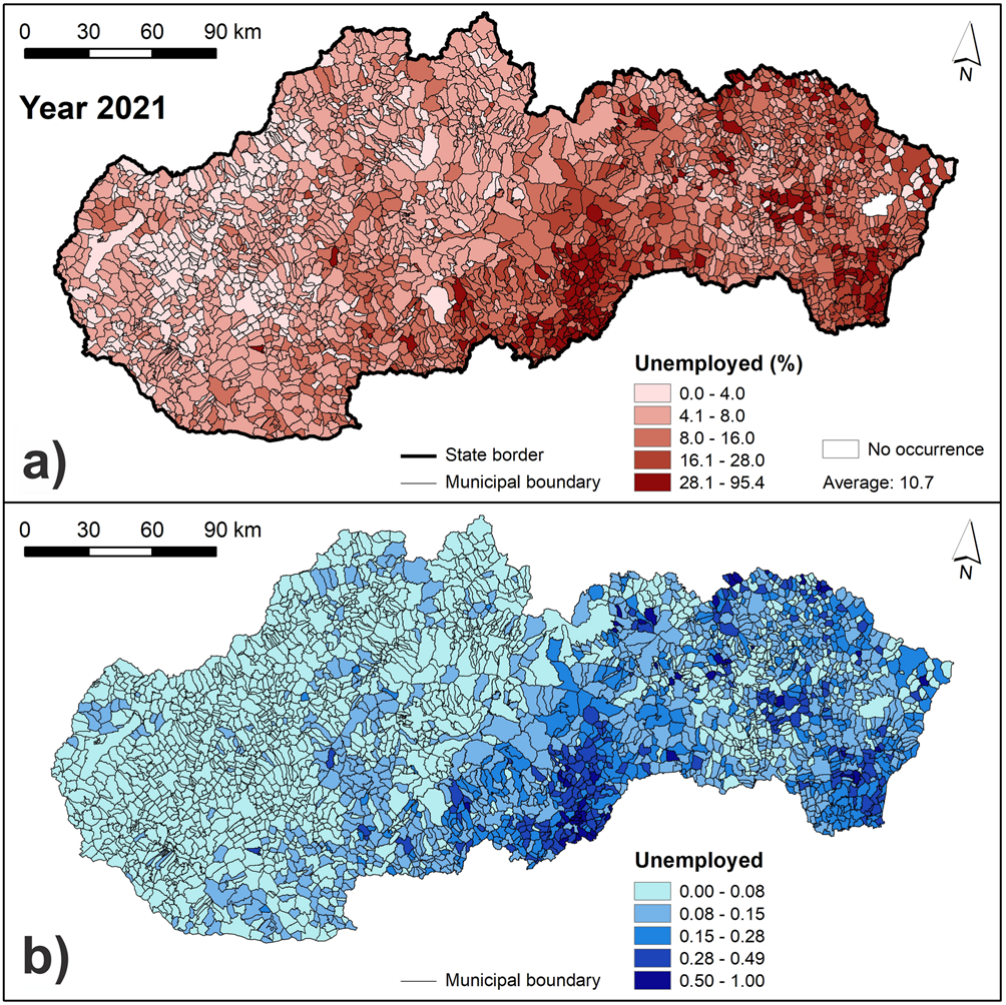
Indicator of the share of unemployed persons from the total number of economically active population: (**a**) original values; (**b**) normalized values. This figure was generated in ArcGIS 10.2.2 software.

The resulting maps of vulnerability indicators are presented in Figs. 7, 8, 9 and 10. Figure 7 presents the number of buildings and the length of roads within 100 m from a river. The highest values of these two indicators can be seen in municipalities located mainly in northern and central Slovakia. Similarly, Fig. 8 shows that the municipalities with the highest number of bridges are located in central Slovakia. As for the population density, the highest values can be seen mainly in larger regional cities and some of the district towns in Slovakia (Fig. 8). Figure 9 presents the share of the Roma ethnicity, which is the highest in municipalities of southern and eastern Slovakia while the share of population aged 65+ is the highest especially in western and also in central Slovakia. The last indicator of the share of unemployed persons from the total number of economically active persons is shown in Fig. 10. The highest values can be seen again in southern and eastern Slovakia, similarly as in case of the Roma ethnicity.

### Weights of indicators

The results of the Pearson correlation between individual hazard and vulnerability indicators and the number of flood events in municipalities is presented in Table 1. In case of the hazard indicators, the highest value was recorded by lithology (0.17) while the lowest value by soil texture (− 0.03). As for the vulnerability indicators, the highest correlation value was recorded by the number of buildings within 100 m from a river (0.29) while the lowest was recorded by the share of unemployed persons from the economically active population of municipality (0.03). The resulting normalized weights correspond to the strength of the Pearson correlation, as can be seen in Table 1.

**Table 1:** Correlation between indicators and the number of flood events and normalized weights of indicators.

Hazard indicator	Pearson correlation	Order of importance (1—the most important indicator; 8—the least important indicator)	Normalized weight (*w*_*j*_)
Number of flood events	–	1	0.222
Lithology (rock permeability)	0.17	2	0.194
River density	0.15	3	0.167
5-day maximum rainfall	0.14	4	0.139
Slope angle	− 0.11	5	0.111
Curvature	0.08	6	0.083
Land cover	− 0.05	7	0.056
Soil texture	− 0.03	8	0.028

Vulnerability indicator	Pearson correlation	Order of importance (1—the most important indicator; 7—the least important indicator)	Normalized weight (*w*_*j*_)
Number of buildings within 100 m from a river	0.29	1	0.250
Number of bridges	0.26	2	0.214
Length of roads within 100 m from a river	0.24	3	0.179
Population 65+	0.13	4	0.143
Population density of urban areas	0.08	5	0.107
Roma ethnicity	0.07	6	0.071
Unemployed	0.03	7	0.036

### Fluvial flood hazard index (FFHI)

**Figure 11:**
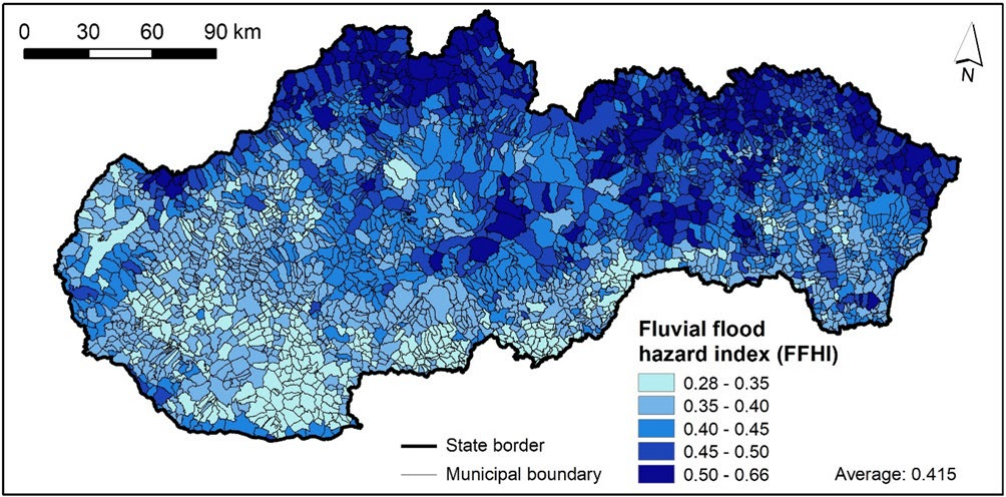
FFHI in municipalities of Slovakia. This figure was generated in ArcGIS 10.2.2 software.

The FFHI values for municipalities are presented in Fig. 11, where the FFHI classes were divided so that the mean value falls within the boundaries of the middle interval. The highest FFHI values were recorded mostly by municipalities in northern and eastern Slovakia and partly also in western and central Slovakia. This is caused, especially, by high number of flood events, unfavorable lithology (like flyschoid rocks) as well as higher river density and rainfall amounts. On the other hand, the lowest FFHI values can be found in municipalities located in western and southern Slovakia, which are characterized by lower occurrence of flood events, permeable lithology or low rainfall amounts. The distribution of municipalities in individual FFHI classes is presented in Fig. 14. The very high class of FFHI includes 306 municipalities, high classes 533 municipalities, moderate class 765 municipalities, low class 886 municipalities, and very low class includes 437 municipalities.

### Fluvial flood vulnerability index (FFVI)

**Figure 12:**
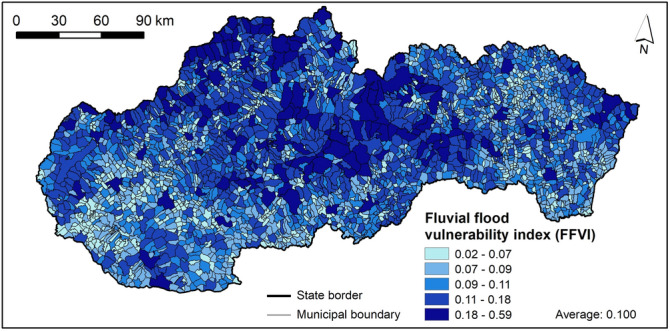
FFVI in municipalities of Slovakia. This figure was generated in ArcGIS 10.2.2 software.

The FFVI values for municipalities are presented in Fig. 12, where the FFVI classes were divided so that the mean value falls within the boundaries of the middle interval. The highest FFVI values were recorded mostly by municipalities in northern and central Slovakia and partly also in western and eastern Slovakia, which is caused mainly by high number of buildings and high length of roads located within 100 m from a river, high number of bridges as well as higher share of population aged 65+. On the contrary, the lowest FFVI values can be found in small-sized municipalities located mainly in western and southern Slovakia, but some also in eastern Slovakia. Although in southern and eastern Slovakia the presence of the Roma ethnicity and unemployed persons is high, the number of buildings and length of roads within 100 m from a river is lower, generally, due the area extent of these municipalities. The distribution of municipalities in FFVI classes is shown in Fig. 14. The very high class of FFVI includes 144 municipalities, high classes 673 municipalities, moderate class 644 municipalities, low class 880 municipalities, and very low class includes 586 municipalities.

### Fluvial flood risk index (FFRI)

**Figure 13:**
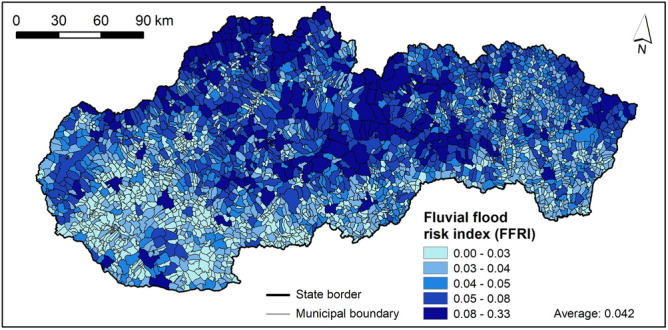
FFRI in municipalities of Slovakia. This figure was generated in ArcGIS 10.2.2 software.

**Figure 14:**
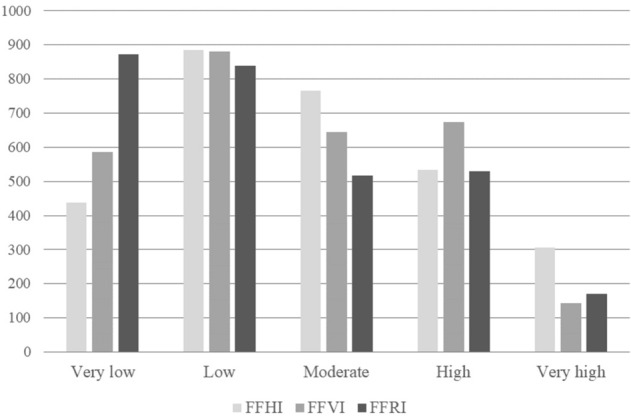
Distribution of municipalities in FFHI, FFVI, and FFRI classes.

The final FFRI values for municipalities are presented in Fig. 13. FFRI classes were classified in the way that the mean value falls within the boundaries of the middle interval. The highest FFRI values were recorded mostly by municipalities in northern, central, and eastern Slovakia and partly also in western Slovakia, where the FFHI and FFVI are relatively high. On the contrary, the lowest FFRI values can be seen in municipalities mainly in western and southern Slovakia with lower values of both FFHI and FFVI. The distribution of municipalities in FFRI classes is shown in Fig. 14. The very high class of FFRI includes 171 municipalities, high classes 529 municipalities, moderate class 517 municipalities, low class 838 municipalities, and very low class includes 872 municipalities. Overall, the FFHI is slightly more dominant in municipalities of Slovakia than the FFVI, as 839 municipalities recorded high and very high FFHI while in case of the FFVI, there were 817 municipalities in high and very high classes.

### Comparison of FFRI and PFRA

**Figure 15:**
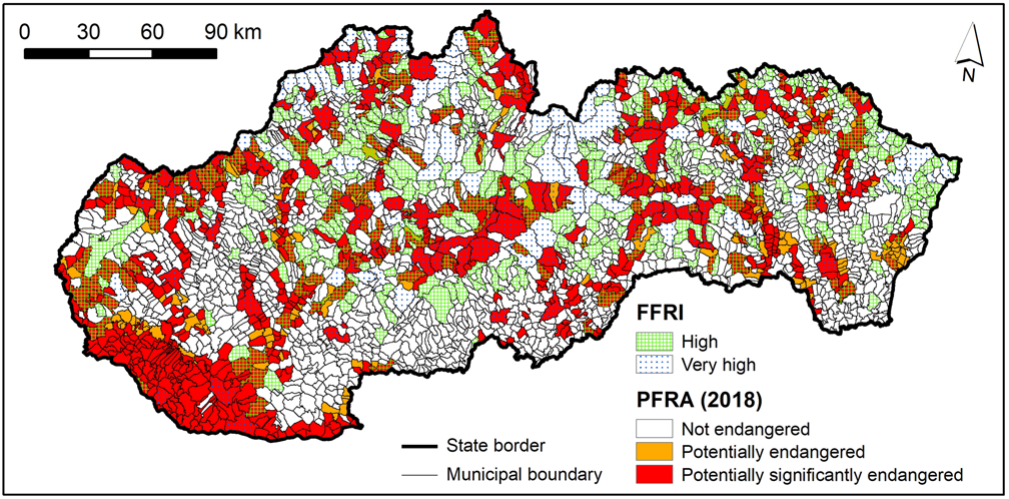
Comparison of FFRI and PFRA^41^ assessment in municipalities. This figure was generated in ArcGIS 10.2.2 software.

The resulting FFRI map was compared with the official PFRA^41^ from 2018. In PFRA^41^, the municipalities were divided into two classes: (1) potentially endangered and (2) potentially significantly endangered. The total number of municipalities labelled as potentially significantly endangered is 722 while the number of municipalities labelled as potentially endangered is 166. Municipalities, which are not listed in the PFRA^41^, and were not labelled as potentially endangered or potentially significantly endangered, were assigned the attribute "not endangered". The information about the inclusion of municipalities in one of the three PFRA^41^ classes (i.e. not endangered, potentially endangered, or potentially significantly endangered) was added to GIS and these three classes were overlain with the calculated FFRI classes for comparison purposes (Fig. 15). The number of matching municipalities is 267, when we compare the potentially significantly endangered class from PFRA^41^ and high and very high FFRI classes. Furthermore, when we compare the potentially endangered municipalities from PFRA with moderate, high, and very high FFRI classes, the number of matching municipalities is 42.

The primary difference between the PFRA^41^ and FFRI is in the methodology applied. In PFRA^41^, the methodology for assigning the municipalities to one of the two classes (potentially endangered or potentially significantly endangered) was based primarily on the information regarding the meteorological and hydrological causes of floods, reports on the course and consequences of floods, and flood potential defined by Minár et al.^53^. The main output was a list of critical river sections and geographical areas, where potentially significant flood risk exists or is likely to occur and also the list of municipalities, which are located along the critical river sections. The selection of the areas with significant or likely occurrence of flood risk was made on the basis of several exclusion criteria.

As a result, we consider the approach applied in the PFRA^41^ insufficient, as no flood hazard or vulnerability index was calculated and, especially, no flood vulnerability assessment was carried out in the PFRA^41^, which is the biggest difference to the approach presented in this study. Therefore, the intended purpose of this study was to propose a comprehensive approach based on detailed and readily available input data and generally agreed concept of flood risk, which is composed of hazard and vulnerability components, and thus provide a reasonable basis for the application of different flood measures and strategies at municipal level.

## Conclusion

In this study, we presented an indicator-based approach for fluvial flood risk mapping and assessment at municipal level in Slovakia. Using eight fluvial flood hazard indicators and seven fluvial flood vulnerability indicators, we computed the FFHI and FFVI. FFRI was determined as a synthesis of FFHI and FFVI.

Based on the results, the very high and high classes of FFHI contain 839 municipalities, which are located mostly in northern and eastern Slovakia and partly also in western and central Slovakia. The primary causes of high and very high FFHI values are in the high number of flood events, unfavorable lithology as well as higher river density and rainfall amounts. As for the FFVI, the very high and high classes include 817 municipalities mainly in northern and central Slovakia and partly also in western and eastern Slovakia. This is caused mainly by high number of buildings and length of roads located within 100 m from a river, high number of bridges, and higher share of population aged 65+. The highest FFRI values were recorded mostly by municipalities in northern, central, and eastern Slovakia and partly also in western Slovakia. The very high and high risk of fluvial flooding is in 700 municipalities, i.e. these municipalities are included in the very high and high classes of FFRI.

Recommendations from this study are, especially, concerned with the applied indicator-based approach to fluvial flood risk mapping, which can be characterized as comprehensive, detailed enough, and using readily available data. These characteristics enable to develop systematical mapping and assessment of flood risk, including the hazard and vulnerability components, at municipal level in Slovakia, which change in space and time over the years.

The results of this study are mainly useful for integrated flood risk management at national scale, where it is possible to prioritize the municipalities with high and very high FFRI in terms of flood measures and strategies. They are also important for local (municipal) scale, which should play a crucial role in the decentralized flood risk management, affecting different aspects of flood prevention, mitigation, protection or adaptation.

As for the future research, we would like to focus on assessing the spatio-temporal development, change, and prediction of FFRI, as well as FFHI and FFVI, and compare these indices within past, present, and future time horizons.

## Supplementary Information

Supplementary Information.

## Data Availability

All of the source data, which support the findings of this article, are publicly available and the weblinks for source data are provided in Supplementary Information. Other requests on data used in this study can be sent to the corresponding author.
