# Supplementary material for: Indicator-based approach for fluvial flood risk assessment at municipal level in Slovakia
Source: Sci Rep. 2023 Mar 27;13:5014. doi: 10.1038/s41598-023-32239-7 (PMC10043001; doi:10.1038/s41598-023-32239-7)
Supplement: Supplementary file 1 — Supplementary Information. [file 41598_2023_32239_MOESM1_ESM.pdf]

Supplementary Information to the paper "Indicator-based approach for fluvial flood risk assessment at municipal level in Slovakia" by Matej Vojtek

Table S1: Source data for fluvial flood hazard indicators and fluvial flood vulnerability indicators.

Fluvial flood hazard indicators				
**Input data**	**Source**	**Format**	**Weblink**	**Indicator**
DEM 10 m (DMR3.5)	Geodetic and Cartographic Institute in Bratislava	Raster	https://www.geoportal.sk/en/zbgis/download/	Slope angle, Curvature
Average annual 5-day maximum rainfall (1981-2010)	Climate Atlas of Slovakia	Map	http://klimat.shmu.sk/kas/	5-day maximum rainfall
Hydrography – river network	Geodetic and Cartographic Institute in Bratislava	Vector	https://www.geoportal.sk/en/inspire/download-services/	River density
CORINE Land Cover 2018	Copernicus Land Monitoring Service (CLMS)	Vector	https://land.copernicus.eu/pan-european/corine-land-cover/clc2018	Land cover
Evaluated soil-ecological units (BPEJ) / Forest soil units	Soil Science and Conservation Research Institute in Bratislava / National Forest Centre in Zvolen	Vector	https://data.gov.sk/en/dataset/bonitovane-podnoekologicke-jednotky-bpej https://data.gov.sk/en/dataset/lesne-podne-jednotky	Soil texture
Map of Engineering Geological Zones 1:50,000	State Geological Institute of Dionýz Štúr in Bratislava	Vector	https://www.geology.sk/maps-and-data/map-services/wms-services/?lang=en	Lithology
Reports on the Course and Consequences of Floods in the Slovak Republic (2001-2021) / Preliminary	Ministry of Environment of the Slovak Republic	Documents	https://www.minzp.sk/voda/ochrana-pred-povodnami/informacie/ https://www.minzp.sk/voda/ochrana-pred-povodnami/manazment-povodnovych-rizik/predbezne-hodnotenie-	Number of flood events

Flood Risk Assessment in the Slovak Republic (2018)			povodnoveho-rizika-2011.html https://www.minzp.sk/voda/ochrana-pred-povodnami/manazment-povodnovych-rizik/predbezne-hodnotenie-povodnoveho-rizika-2018.html	
**Fluvial flood vulnerability indicators**				
**Input data**	**Source**	**Format**	**Weblink**	**Indicator**
ZBGIS (2021)	Geodetic and Cartographic Institute in Bratislava	Vector	https://zbgis.skgeodesy.sk/tkgis/default.aspx	Number of buildings within 100 m from a river, Length of roads within 100 m from a river, Number of bridges
Data cubes (2021)	Statistical Office of the Slovak Republic	Spreadsheet	http://datacube.statistics.sk	Population density of urban areas, Population 65+, Unemployed
Atlas of Roma Communities (2019)	VEDA	Spreadsheet	https://www.romovia.vlada.gov.sk/atlas-romskych-komunit/atlas-romskych-komunit-2019/?csrt=6077692989717954430	Roma ethnicity

Table S2: Classification of hazard indicators, importance and normalized weights of indicator classes.

**Slope (°)**<br>Classification: Demek (1972)	**Order of importance (1 – the most important indicator; 5 – the least important indicator)**	**Normalized weight (w_j)**
<2.0	1	0.333
2.0-5.0	2	0.267
5.0-15.0	3	0.200
15.0-35.0	4	0.133
35.0<	5	0.067
**Curvature**	**Order of importance (1 – the most important indicator; 3 – the least important indicator)**	**Normalized weight (w_j)**
Concave	1	0.500
Linear	2	0.333
Convex	3	0.167
**Lithology (rock permeability)**<br>Classification: Hrnčiarová (1993)	**Order of importance (1 – the most important indicator; 5 – the least important indicator)**	**Normalized weight (w_j)**
Very low permeability	1	0.333
Low permeability	2	0.267
Moderate permeability	3	0.200
High permeability	4	0.133
Very high permeability	5	0.067
**Soil texture**<br>Classification: Linkeš et al. (1996)	**Order of importance (1 – the most important indicator; 6 – the least important indicator)**	**Normalized weight (w_j)**
Clayey and clay	1	0.286
Clayey-loamy	2	0.238
Loamy	3	0.190
Sandy-loamy	4	0.143
Loamy-sandy	5	0.095
Sandy	6	0.048
**CORINE Land Cover 2018**	**Order of importance (1 – the most important indicator; 13 – the least important indicator)**	**Normalized weight (w_j)**
11 Urban fabric	1	0.143
12 Industrial, commercial and transport units	2	0.132
13 Mine, dump and construction sites	3	0.121
33 Open spaces with little or no vegetation	4	0.110
14 Artificial, non-agricultural vegetated areas	5	0.099

21 Arable land	6	0.088
22 Permanent crops	7	0.077
24 Heterogeneous agricultural areas	8	0.066
23 Pastures	9	0.055
32 Scrub and/or herbaceous vegetation associations	10	0.044
31 Forests	11	0.033
41 Inland wetlands	12	0.022
51 Inland waters	13	0.011
**5-day maximum rainfall (mm)**	**Order of importance (1 – the most important indicator; 5 – the least important indicator)**	**Normalized weight (w_j)**
90<	1	0.333
70-90	2	0.267
60-70	3	0.200
50-60	4	0.133
<50	5	0.067
